# Is the antepsoas oblique lumbosacral interbody fusion safe in patients with aortoiliac calcification?

**DOI:** 10.1016/j.xnsj.2026.100867

**Published:** 2026-02-13

**Authors:** Chadi Tannoury, Hayley Denwood, Rehan R. Khan, Rutvin J. Kyada, Olivia T. Zhou, Sara Atassi, Aziz Saade, Mirna N. Chahine, Tony Tannoury

**Affiliations:** a Department of Orthopedic Surgery, Boston Medical Center, Boston, MA, United States; b Boston University Chobanian & Avedisian School of Medicine, Boston, MA, United States; c NYU Langone Orthopedic Hospital, NYU Langone Health, New York, NY, United States; d University of Balamand, Faculty of Arts & Sciences, Department of Biology, Dekwaneh Campus, Sin el fil, Lebanon; e Foundation Medical Research Institutes (F-MRI) (R), Geneva, Switzerland

**Keywords:** Lumbosacral spine, Minimally invasive antepsoas, ATP, OLIF, ALIF, Aortoiliac calcification, Lumbar fusion, Vascular injury

## Abstract

**Background:**

The anterior approaches to lumbar arthrodesis, including direct anterior (ALIF) and antepsoas (ATP)/oblique (OLIF) fusions, require careful manipulation of the abdominal prevertebral vessels for safe and adequate spinal access. Therefore, surgeons who perform anterior lumbar fusions in patients with aortoiliac calcifications are often cautious due to concerns for perioperative vascular complications, as well as the associated risks of concomitant medical comorbidities. This study sought to compare the incidence of perioperative vascular and medical complications in patients with and without abdominal aortoiliac calcification (AAC) undergoing the minimally invasive antepsoas (MIS-ATP) lumbosacral fusion.

**Methods:**

This was a retrospective matched cohort study including 482 adult patients undergoing MIS-ATP lumbosacral fusions at a single institution between 2014 and 2020 (227 with AAC and 255 without AAC), matched by sex and American Society of Anesthesiologists (ASA) scores. Through preoperative standing lateral lumbar radiographs, using Kaupilla et al.’s AAC grading system, we graded anterior and posterior aortic wall calcification from L1 to L4. Electronic medical records were reviewed to identify and collect the perioperative complications.

**Results:**

While there was no occurrence of intraoperative vascular injuries in either group, patients with AAC were more likely to develop medical complication (34.8% vs. 13.3%, p < .001), with anemia (18.9% vs. 9.2%), ileus (16.3% vs. 2.7%) and acute kidney injury (6.6% vs. 5.5%) being the most common. Overall, individuals with AAC had 2.62 times increased odds of developing a postoperative medical complication. Moreover, moderate AAC was found to be a significant risk factor (OR = 3.48).

**Conclusions:**

Presence of AAC in patients undergoing MIS-ATP fusion was not associated with increased risk of direct surgical or exposure related vascular complications. However, patients with AAC were significantly more likely to experience medical complications following MIS-ATP fusion.

## Introduction

In the recent decade, rapid advancements in the field of minimally invasive spine surgery have transformed the treatment of adult spinal disorders [1]. Anterior approaches to the lumbar spine have shown benefits of direct access to the intervertebral disc spaces allowing for anterior column release, decompression, and reconstruction [2]. Safe anterior access to the lumbar spine is crucial for mitigating complications, and is predicated on a judicious vascular handling, often necessitating the assistance of access surgeons [3]. Beyond access related retractions, the prevertebral vessels can be further stretched following anterior lumbar column reconstruction and lordosis restoration. Accordingly, vascular complications have been reported in anterior lumbar fusions, ranging widely from 1.4% to 24% [4,5]. Even though vascular injury rates have been historically derived from direct anterior (ALIF) exposure, the minimally invasive antepsoas (MIS-ATP) technique uses an anterior oblique corridor and requires relatively less extensive manipulation of the prevertebral vessels. Moreover, calcification of the prevertebral vessels remains a debatable risk factor for complications, and a concerning radiographic finding when contemplating an anterior approach to the spine [6,7].

The minimally invasive antepsoas (MIS-ATP) technique is a subset of anterior lumbar interbody fusions that, comparable to the oblique lumbar interbody fusion (OLIF) technique, utilizes an oblique retroperitoneal psoas-preserving access to the lumbar discs anywhere between T12-S1 [8]. Advantages of the MIS-ATP technique, in well trained hands, include providing direct visualization of the prevertebral vessels including the vena cava, abdominal aorta, and iliac vessels, and therefore the ability to safely retract and protect them [8]. Unlike traditional ALIF, MIS-ATP approaches the spine through an oblique retroperitoneal window and requires less extensive mobilization of the major abdominal vessels, which fundamentally changes the mechanism and likelihood of exposurerelated vascular injury. In previous reports, when compared to other anterior lumbar approaches, the MIS-ATP technique exhibited a significantly lower overall complication rate (8.2%) and no major direct vascular injuries [9]. The aim of this study is to evaluate the relevance of aortoiliac calcification in patients undergoing MIS-ATP fusion, and the associated risk of developing adverse events.

## Methods

### Study design

Following an institutional Review Board (IRB) approval, a retrospective chart review of patients who underwent the minimally invasive antepsoas fusion (MIS-ATP) complemented by posterior percutaneous fusion (PPF) at our academic center between 2014 and 2020 was conducted. Inclusion criteria were the following: (1) adult patients with spinal disease; (2) underwent the MIS-ATP + PPF of the lumbosacral spine; (3) preoperative lateral standing x-ray within 0 to 12 months of their operation; and (4) treatment at our institution.

We reviewed the radiology reports for 914 patients. Fifty-five patients were excluded from undergoing a procedure other than MIS-ATP. Additionally, 169 patients were excluded for either having preoperative x-rays taken ≥ 1 year prior to surgery, inaccessible preoperative imaging, or unavailable standing lateral x-rays.

Radiology reports and imaging were reviewed to confirm the presence, or lack of abdominal aortoiliac calcification (AAC). We identified 227 adult patients with radiographic AAC who underwent MIS-ATP + PPF. Of the remaining 463 patients who underwent MIS-ATP fusion and did not have AAC, a cohort of 255 patients without AAC were matched with the calcification group by sex and morbidity using the American Society of Anesthesiology (ASA) class.

The 2 cohorts of patients, those in the AAC and no-AAC groups, were matched exclusively on sex and American Society of Anesthesiologists (ASA) class; no additional variables, including demographic or medical comorbidities, were included in the matching process for clinically important reasons. A limited matching framework where patients were matched only on their sex and ASA class was utilized for a few reasons. From an analysis standpoint, fully matching patients across all comorbidities (eg, hypertension, diabetes, hyperlipidemia, coronary artery disease, tobacco use, etc.) would not be feasible in retrospective AAC research as it would essentially eliminate the true AAC phenotype, which contains these inherent properties. However, to control for these important risks between the 2 cohorts, baseline differences were adjusted for using multivariate regression modelling (standard analytic approach in AAC studies) to control for confounders.

### Demographics and clinical variables

Demographic characteristics collected were age at time of surgery, sex, race, body mass index (BMI), tobacco use, and alcohol use. Past medical history collected included coronary artery disease (CAD), hypertension, history of myocardial infarction, history of stroke, hyperlipidemia/hypercholesterolemia, diabetes mellitus, and chronic kidney disease (CKD).

Operative variables collected included the number of anterior surgical levels, and intraoperative abdominal vascular injuries/events. Surgical extent was included as a covariate in all multivariable analyses to account for any potential confounding related to construct length variations.

Perioperative complications collected were: postoperative vascular (ie, abdominal, iliac, or peripheral) events, deep vein thrombosis (DVT), pulmonary embolism (PE), limb ischemia, mesenteric vascular events, myocardial infarction (MI), stroke, acute kidney injury (AKI), retroperitoneal hematoma, anemia, and ileus. Complications were analyzed individually and as a binary categorical variable. Complications were meticulously collected using standardized clinical documentation within the electronic medical record of the present study’s hospital system, during either the index hospitalization or in the perioperative period. Complications were analyzed individually as well as composite outcomes.

### Grading system

**Fig. 1:**
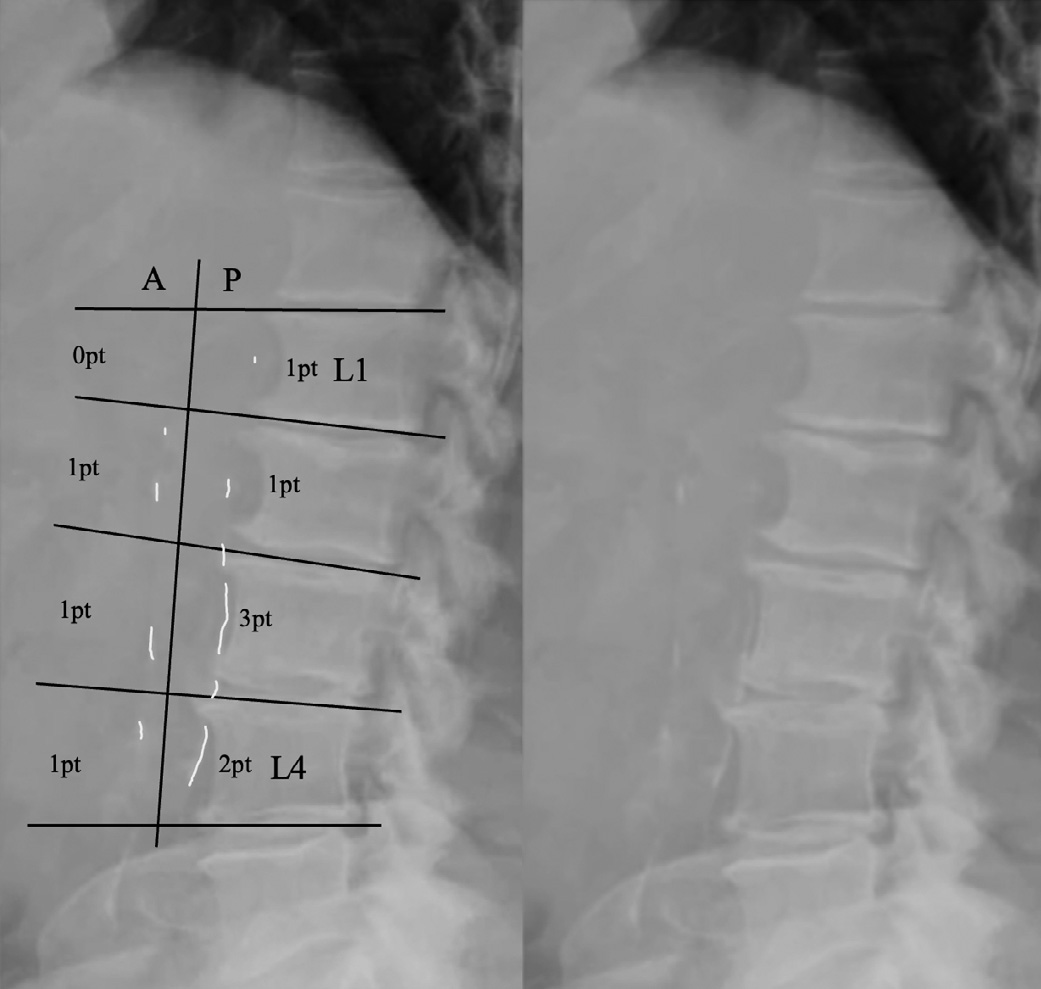
Example of grading of abdominal aortic calcification per Kauppila's scoring system [11].

**Fig. 2:**
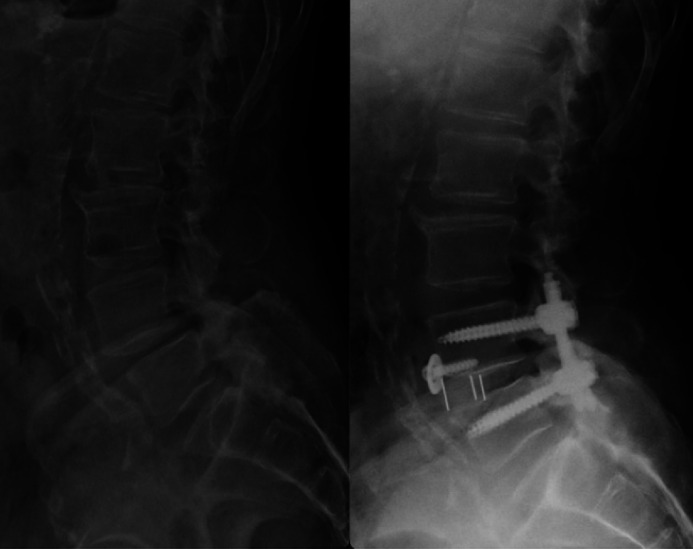
Pre and postoperative lateral X-ray of a patient with AAC that underwent MIS-ATP lumbosacral fusion.

The grading system created by Kauppila et al. [10] was utilized to evaluate the presence and extent of AAC. A preoperative standing lateral lumbar spine X-ray taken within 1 year of the operation was used for grading. For each segment between L1 to L4, both the anterior and posterior wall of the aorta were graded for the extent of calcification. Severity was given points based on the following: 0 points: No calcific deposits; 1 point: less than 1/3 of the wall calcified; 2 points: between 1/3 and 2/3 of the wall affected; 3 points: more than 2/3 of the wall affected (Fig. 1). The final score was the sum of the anterior and posterior points between L1 to L4. Scores ranged between 0 and 24, with higher values indicating more extensive calcification. Each patient received a total of 2 grades from 2 out of 3 independent graders (HD, RK, OZ). The mean AAC was used for statistical analysis. Inter-rater reliability was assessed with an intraclass correlation coefficient (ICC), which was 0.98. Inter-reliability for grading of specific levels was also fairly high with ICC ranging from 0.883 to 0.926. Each patient was designated an AAC severity score defined by literature cut-offs, which was therefore defined as: low (0–1), moderate (2–5), or extensive (≥6) [10]. A preoperative and postoperative standing lateral lumbar spine x-ray of one of the patients with AAC can be seen in Fig. 2.

### Statistical analysis

Continuous variables were compared using an independent 2 sample t-test. Categorical variables were analyzed using the Chi-square test. A simple logistic regression analysis was used to assess an association between any presence of AAC and any perioperative complication. Multivariable logistic regression analysis was used to assess an association between AAC severity and any perioperative complication. A p ≤ .05 was considered statistically significant. Regression analyses controlled for age, sex, BMI, ASA, race/ethnicity, number of anterior levels operated upon, diabetes, hypertension, chronic kidney disease, hyperlipidemia, smoking status, alcohol use, CAD history, and stroke history. All analyses were performed using SPSS software version 25.0 (IBM, Armonk, NY, USA). To prevent overmatching, eliminating the AAC phenotype altogether, and losing the sample size, fully matching across all baseline comorbidities was intentionally avoided. However, all baseline risks between the AAC and non-ACC cohorts were controlled for in the multivariable regression model.

Additional stratified analyses were performed by number of fused levels (≤2 vs. ≥3) to address potential confounding related to surgical extent. Stratified models using AAC severity categories (low, moderate, extensive) were not feasible in the ≤2 level group due to small sample sizes; even univariate models did not converge. To enable convergence in stratified models, covariates were reduced by condensing comorbidities into a single binary variable (≥1 vs. none); smoking and alcohol were merged into a single binary substance use variable, and race into a binary White versus non-White variable.

## Results

### Cohort characteristics

This retrospective matched cohort study included 482 patients: 227 subjects with AAC, and 255 without (No AAC). The average age for the entire cohort was 57.7 ± 11.6 years, the average BMI was 31.2 ± 1.63 kg/m^2^, and 57.7% were female (Table 1). There was no significant difference between ASA class (2.53 vs. 2.51, p = .603) and sex (50.2% vs. 50.2%, p = .996) between the AAC and No-AAC groups. The moderate AAC group consisted of 46.7% patients with AAC, followed by extensive (42.7%) and low (10.6%) AAC.

**Table 1:** Descriptive characteristics of the included cohort stratified by the complication and no complication groups.

	Total cohort (*N* = 482)	No calcification (*N* = 255)	Calcification (*N* = 227)	p-value
Demographics				
Age (yrs)	57.7 ± 11.6	53.1 ± 11.3	62.8 ± 9.6	<.001
Females	242 (50.2%)	128 (50.2%)	114 (50.2%)	.996
Race				
White	250 (51.9%)	119 (46.7%)	131 (57.7%)	.039
Black	98 (20.3%)	56 (22.0%)	42 (18.5%)	
Hispanic	73 (15.1%)	48 (18.8%)	25 (11.0%)	
Other/Unreported	61 (12.7%)	32 (12.5%)	29 (12.8%)	
Operative variables				
ASA score	2.52 ± 0.52	2.51 ± 0.51	2.53 ± 0.53	.603
Number of levels operated upon	3.66 ± 1.63	3.45 ± 1.63	3.90 ± 1.59	.002
Lifestyle factors				
Former/current tobacco use	253 (52.5%)	103 (40.4%)	150 (66.1%)	<.001
Former/current alcohol use	163 (33.9%)	90 (35.3%)	73 (32.3%)	.489
BMI (kg/m^2^)	31.2 ± 6.2	31.4 ± 6.2	31.0 ± 6.2	.421
Comorbidities				
Hypertension	278 (57.8%)	116 (45.5%)	162 (71.7%)	<.001
Diabetes	117 (24.3%)	48 (18.9%)	69 (30.4%)	.003
Chronic kidney disease	46 (9.5%)	19 (7.5%)	27 (11.9%)	.097
Hyperlipidemia	209 (43.4%)	87 (34.1%)	122 (53.7%)	<.001
Medical history				
History of CAD	49 (10.2%)	12 (4.7%)	37 (16.3%)	<0.001
History of stroke	35 (7.3%)	14 (5.5%)	21 (9.3%)	0.112
Perioperative complications				
Mean number of complications	0.34 ± 0.74	0.20 ± 0.60	0.49 ± 0.84	<0.001
Any complication	113 ± 23.4	34 ± 13.3	79 ± 34.8	<0.001
DVT	4 (0.8%)	1 (0.4%)	3 (1.3%)	
PE	7 (1.5%)	1 (0.4%)	6 (2.6%)	
Limb ischemia	0 (0.0%)	0 (0.0%)	0 (0.0%)	
Mesenteric vascular event	0 (0.0%)	0 (0.0%)	0 (0.0%)	
MI/demand ischemia	7 (1.5%)	2 (0.8%)	5 (2.2%)	
AKI	29 (6.0%)	14 (5.5%)	15 (6.6%)	
Retroperitoneal hematoma	4 (0.8%)	2 (0.8%)	2 (0.9%)	
Anemia	66 (13.7%)	23 (9.0%)	43 (18.9%)	
Requiring transfusion	53 (11.0%)	21 (8.2%)	32 (14.1%)	
Ileus	44 (9.1%)	7 (2.7%)	37 (16.3%)	

ASA, American Society of Anesthesiologists; BMI, body mass index; CAD, coronary artery disease; DVT, deep vein thrombosis; PE, pulmonary embolism; MI, myocardial infarct; AKI, acute kidney injury.

Patients with AAC were more likely to be older (62.8 vs. 53.1 years, p < .001), white (57.7% vs. 46.7%, p = .039), and have a higher number of surgical spinal levels (3.90 vs. 3.45, p = .002) (Table 1). Patients in the AAC group were also more likely to be current or former smokers (66.1% vs. 40.4%, p < .001), have medical and metabolic comorbidities, including hypertension (71.7% vs. 45.5%, p < .001), diabetes mellitus (30.4% vs. 18.9%, p = .003), hyperlipidemia (54.7% vs. 34.1%, p < .001), and coronary artery disease (16.3% vs. 4.7%, p < .001).

### Perioperative complications and vascular events

The AAC group was noted to have a higher mean number of medical complications (0.20 vs. 0.49, p < .001) and was significantly more likely to experience ≥1 complication (p < .001). Irrespectively, there were no cases of acute limb ischemia, intraoperative vascular injuries, or mesenteric vascular events in either group. In the AAC group, 6 patients had a pulmonary embolism (2.6%) and 5 patients had a myocardial infarction (2.2%). The most common medical complications in patients with AAC were postoperative anemia (18.9%), ileus (16.4%), and AKI (6.6%) compared to their matched No-AAC cohort.

### Association between AAC and postoperative complications

When controlling for confounders, individuals with AAC had 2.62 times increased odds of developing a postoperative complication (OR 2.62, 95% CI 1.45–4.74, p < .001). When stratifying the calcification group by severity, individuals with moderate AAC had 3.48 times increased odds of experiencing a postoperative complication (OR 3.48, 95% CI 1.79–6.77, p < .001). Despite the low and extensive AAC groups having increased odds of complication in simple logistic regression, controlling for confounders suggested no clear association (Table 2).

**Table 2:** Simple and multivariable logistic regression analysis testing the association between the degree of abdominal aortic calcification and the odds of having a perioperative complication.

Exposure variables	Simple OR (95% CI)	p-value	Adjusted OR (95% CI)	p-value
Any calcification	3.47 (2.21–5.46)	<.001	2.62 (1.45–4.74)	.001
AAC severity categories (Reference = no calcification cohort)				
Low (0–1)	2.17 (0.80–5.84)	.127	1.75 (0.54–5.71)	.351
Moderate (2–5)	3.94 (2.31–6.72)	<.001	3.48 (1.79–6.77)	<.001
Extensive (≥6)	3.35 (1.93–5.83)	<.001	1.88 (0.87–4.02)	.106

### Influence of surgical extent

We further stratified patients based on the number of fused levels (Table 3). Among patients undergoing 2 or fewer levels of fusion (*N* = 124), presence of any AAC was strongly associated with postoperative complications (adjusted OR 5.39, 95% CI 1.33–21.90, p = .019). While among patients with 3 or more fused levels (*N* = 358), the association between AAC and complications did not reach statistical significance, though a consistent trend toward increased complications persisted (adjusted OR 1.78, 95% CI 0.99–3.18, p = .053) - suggesting that the effect of AAC on complications may be more pronounced in shorter-segment fusions with a less clear relationship in longer constructs.

**Table 3:** Multivariable logistic regression stratified by number of levels fused.

Two or fewer levels fused (*N* = 124)				
Exposure variables	Simple OR (95% CI)	p-value	Adjusted OR (95% CI)	p-value
Any calcification	6.81 (2.09–22.19)	.001	5.39 (1.33–21.90)	.019

Three or more levels fused (*N* = 358)				
Any calcification	2.92 (1.78–4.80)	<.001	1.78 (0.99–3.18)	.053

## Discussion

### Aortoiliac calcification and vascular risk in anterior spine surgery

Atherosclerotic vascular disease leads to a stiffening and reduced elasticity of the prevertebral vessels, potentially predisposing patients to a higher rate of vascular complications during access related anterior vascular manipulation and retraction [11]. Atherosclerotic disease is prevalent among individuals with medical comorbidities including advanced age, hypertension, smoking, dyslipidemia, diabetes mellitus, high BMI, and end stage renal disease (ESRD) [12,13].

Furthermore, atherosclerotic disease is a major risk factor for development of aortic abdominal calcification (AAC), a systemic marker for atherosclerosis. In a study done by Rahman and colleagues, the national prevalence of AAC among persons 40 years and older was found to be 28.8% [14]. With the aging population and increasing obesity rates, many patients that will require spine surgery may fall under this statistic.

### Vascular safety of the MIS-ATP approach

Despite almost 500 MIS-ATP surgical fusion cases in the present study, no intraoperative vascular injuries or acute limb ischemic events were found, even in those patients with moderate or extensive AAC. These specific results highlight the anatomical distinction of the MIS-ATP surgical corridor for interbody fusions—allowing for safe vessel mobilization under direct visualization. The senior authors have developed and gained extensive experience in the MIS-ATP approach, and therefore access surgeons, despite being available to assist, were not involved in any of these cases.

**Fig. 3:**
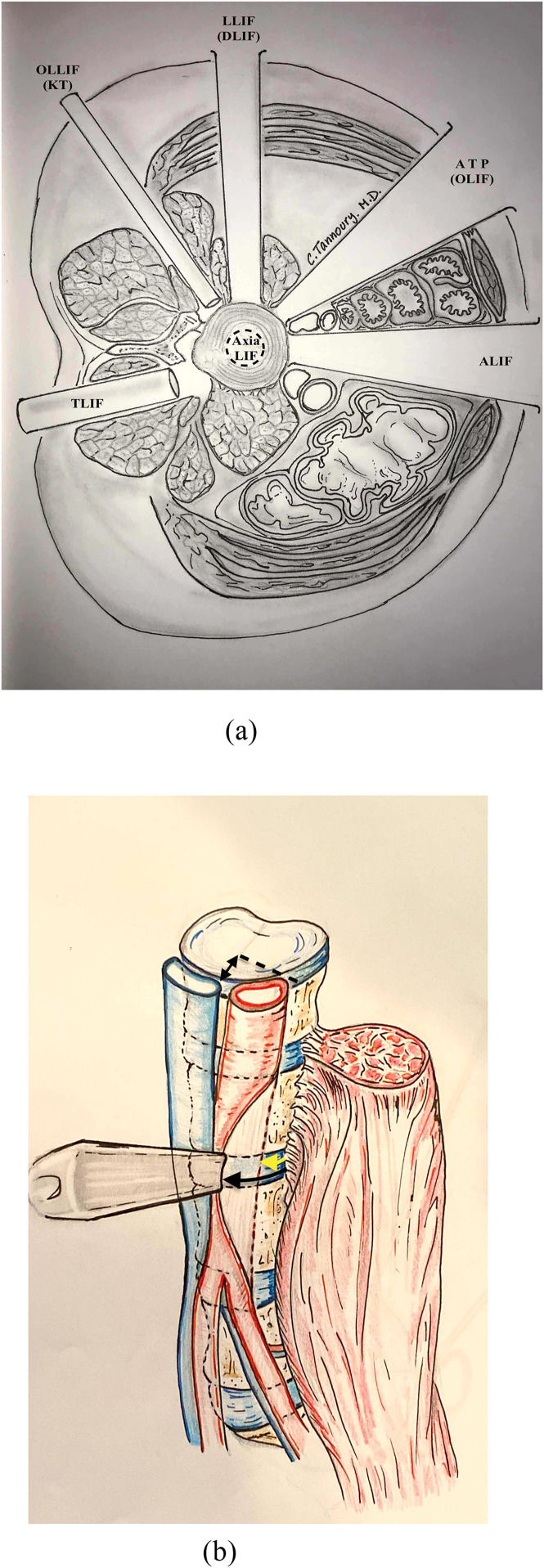
(A) A cross-sectional schematic illustrating the anatomical corridors of common lumbar interbody fusion approaches, including TLIF (Transforaminal Lumbar Interbody Fusion), OLLIF - KT (Oblique posterolateral Lumbar Interbody Fusion - Kambin Triangle), LLIF-DLIF (Lateral Lumbar Interbody Fusion - Direct Lateral Interbody/Transpsoas Fusion), ATP-OLIF (Antepsoas - Oblique Lumbar Interbody Fusion), ALIF (Anterior Lumbar Interbody Fusion), and AxiaLIF (Axial Lumbar Interbody Fusion), in relation to surrounding musculature and visceral structures. Illustration created by an author of the present study and adapted from Tannoury [42]. (B) A schematic illustrating the anatomical antepsoas (ATP) surgical corridor to the lumbar spine developed following prepsoas vascular mobilization. Illustration created by an author of the present study and adapted from prior anatomical and radiographic investigations of the antepsoas surgical corridor [8].

The anatomical surgical corridors, and their relationship to the prevertebral vessels are illustrated in Fig. 3.

### Scope and novelty

This study analyzed whether patients with AAC were at a higher risk of vascular injury and/or medical complications following the MIS-ATP lumbosacral fusion. To our knowledge, this is the largest and only matched cohort focusing on complication risks in patients with AAC undergoing MIS-ATP surgeries. Additionally, there is no existing literature investigating a scoring system quantifying the degree of aortoiliac calcification in patients undergoing the MIS-ATP fusion. Despite manipulating calcified vessels and elongating the anterior spinal column, we encountered no direct vascular injuries or intraoperative vascular events in patients with AAC who underwent MIS-ATP fusions. This is comparable to previous reports on the MIS-ATP technique claiming no major direct vascular injuries [9].

### Comparison with prior literature

The relationship between prevertebral vascular calcification and injuries during anterior spinal surgery remains a topic of debate [6,7,[15], [16], [17], [18], [19]]. Similar to our findings, Garg et al. [7] did not find a clear association between aortoiliac calcification and risk of vascular injury or increased EBL in a cohort of 228 patients who underwent ALIF. In contrast, Rothenfluh et al. [6] found that 25% of patients with calcification developed vascular injury, which was twice as high as the rate of the whole series, but due to the small population size and advanced age, a clear and strong association between AAC and vascular injury was challenged. Also, in contrast to the MIS-ATP approach, and other oblique retroperitoneal approaches, traditional open ALIF requires greater retraction of the great vessels and their branches as they are in the direct path of access to the intervertebral disc [20].

The literature surrounding complications from direct ALIF techniques provides valuable insight into the mechanisms of vascular injury during anterior exposures, highlighting the hazards inherent to the direct mobilization of the major prevertebral vessels. The MIS-ATP technique, albeit an anterior interbody fusion technique, relies on an oblique prepsoas trajectory that takes advantage of the natural prepsoas window obviating the need for excessive great-vessel retraction [8]. These differences in exposure between direct ALIF and the oblique ATP approach are likely accountable for the lower vascular-injury rates observed in our study, and other MIS-ATP series, including patients with advanced AAC.

### Clinical implications of AAC in MIS-ATP fusion

Brau et al. [4] showed that the majority (92%) of the complications arose from vessel retraction during ALIF procedures. Similarly, Garg et al. [7] cautioned about vascular retraction related injuries in patients with increased BMI, male sex, and L4-S1 surgical levels. However in our study, despite the presence of a wide spectrum of radiographic calcification, employing the ATP technique with a careful prevertebral vascular exposure and mobilization has shown to be strongly protective against direct vascular injuries in all patients irrespective of their gender (50.2% with AAC vs. 50.2% without AAC, p = .996), BMI (31.0 kg/m^2^ with AAC vs. 31.4 kg/m^2^ without AAC, p = .421), or surgery involving the L4–S1 levels (96.5% with AAC vs. 97.8% without AAC, p = .399) [[6], [7], [8], [9],^21^]. On the other hand, our findings highlight the relevance of AAC as a systemic risk factor for developing medical complications that may ultimately affect postoperative outcomes.

### Influence of age on MIS-ATP fusion outcomes in patients with AAC

By using a national database with 8,459 patients who underwent ALIF, Li et al. found that increasing age was associated with postoperative complications such as pneumonia, sepsis, and other major complications [22]. Similarly, age and AAC have been found to be associated with poor outcomes in patients undergoing posterior lumbosacral fusion [23,24].

In this investigation, the calcification group had more comorbidities and a significantly higher average age compared to the no calcification group, however on regression analysis, age was not found to be an independent predictor of postoperative complications. Nonetheless, as calcification relates to increasing age, chronic kidney disease, diabetes mellitus, hypertension, and numerous other factors, this complex relationship should be strongly considered during preoperative decision-making and patients’ counseling [12].

### AAC as a risk factor for pulmonary embolism (PE) and deep vein thrombosis (DVT) following MIS-ATP fusion

In our study, patients with AAC were at higher risk of developing PE (2.6% vs. 0.4%) and DVT (1.3% vs. 0.4%). In 2 studies comparing the anterior and posterior lumbar fusion approaches, patients undergoing the anterior approach were more likely to develop postoperative DVT, with an incidence ranging from 1% to 2.2% [25,26]. Contrastly, these studies found no difference in the incidence of PE in patients undergoing either anterior or posterior approach. Our findings suggest that patients with AAC may be at a higher risk of postoperative PE and DVT following anterior MIS-ATP lumbar fusion. One might conclude that the presence of AAC is an indirect indicator of concomitant impairment of the venous circulation, predisposing to DVT and PE.

### AAC as a risk factor for acute cardiac events following MIS-ATP fusion

We found that patients with AAC were at a higher risk of developing myocardial infarction (2.2% with AAC vs. 0.8% no AAC). Previous literature states that the incidence of cardiac complications following ALIF ranges from 0.2 to 0.8%, which is similar to the no calcification group in this study (0.8%) [[26], [27], [28]]. In a cohort of 901 patients undergoing ALIF or TLIF, 7 patients had cardiac complications [29]. Of those 7 patients, there were similar comorbidities found such as hypertension, diabetes mellitus, and presence of aortoiliac calcification. Given the findings of this study and supporting evidence from previous literature, providers should be mindful of the plausible association between AAC and other systemic comorbidities that elevate the risk of postoperative cardiac events in patients undergoing MIS-ATP.

### AAC as a risk factor for acute kidney injury (AKI) following MIS-ATP fusion

This study we found that patients with AAC were more likely to have chronic CKD at baseline (11.9% vs. 7.5%), and subsequently had higher rates of AKI (6.6% vs. 5.5%) following surgery. In a large database study comparing outcomes following ALIF/DLIF (*N* = 2,372) and TLIF/PLIF (*N* = 5,563), there was no difference in acute renal failure (0.04% in ALIF/DLIF vs. 0.1% in TLIF/PLIF) or progressive renal insufficiency (0.2% vs. 0.1%) between groups [26]. Our study observed higher AKI rates (7.5% vs. 0.04%) compared to Shillingford et al., [26] potentially due to 7.5% of our cohort having had baseline CKD, versus 0.1% requiring dialysis in their cohort. Furthermore, cardiometabolic conditions are strongly linked to postoperative AKI in the orthopedic literature, which is further supported by this study [30,31].

### AAC as a risk factor for perioperative anemia and blood transfusion following MIS-ATP fusion

We found that patients with AAC were more likely to develop postoperative anemia (18.9% vs. 9.0%) and were more likely to receive blood transfusions (14% vs. 8.2%). Shillingford et al.s [26] found that patients undergoing PLIF/TLIF had a significantly higher rate of requiring postoperative blood transfusions compared to those undergoing ALIF/DLIF (9.6% vs. 7.6%), but this difference was muted when controlling for covariate. They suggested that patients with higher ASA classes, increased age, preoperative anemia, and bleeding disorders were at a higher risk of requiring a postoperative transfusion regardless of the surgical approach. Our study underscores the increased incidence of anemia in patients with AAC, a finding that can be related to the larger surgical extent in the AAC cohort (3.9 levels vs. 3.4 levels) as well as the associated medical morbidities (ie, kidney disease) which may also affect erythropoiesis [32,33]. Retroperitoneal hematoma was an equally rare finding (0.8%–0.9%) in both AAC and non-AAC cohorts, and therefore it is not thought to be a common risk factor for anemia in patients treated with MIS-ATP fusions. Finally, in our study, patients with AAC were more likely to receive a postoperative blood transfusion (14%) compared to those with No-AAC (8.2%). We presuppose that patients with AAC, who tend to have more medical and cardiac morbidities, may have preferentially received blood transfusion as a resuscitation strategy, to optimize systemic oxygen carrying capacity, rather than the less effective fluid resuscitation which may lead to fluid overload and dilutional anemia.

### AAC as a risk factor for ileus following MIS-ATP fusion

In our study, postoperative ileus (16.3% vs. 2.7%) was more frequently encountered in patients with AAC. Due to the degree of bowel manipulation, ALIF and OLIF (7.5% and 7.2%) are reported to have a higher incidence of postoperative ileus compared to PLIF and LLIF (2.6% and 7.0%) [[34], [35], [36]]. Commonly reported risk factors for ileus include anemia, multilevel fusion, and electrolyte imbalances [37,38]. Although not previously reported in spine literature, patients undergoing colorectal surgery with AAC scores exceeding the calculated optimal cut-off value of 10, experienced a 17.4-fold increase in postoperative complications [39]. Our study supports their findings, highlighting the increased risk of postoperative complications in patients with AAC undergoing MIS-ATP. In patients with AAC, these complications, including anemia, CKD, and AKI-related electrolyte imbalance, may have collectively impacted the development of postoperative ileus.

### Does the extent of radiographic AAC matter?

In this investigation, the grading system created by Kauppila et al. [10] was utilized to evaluate the degree of AAC. Patients with at least some calcification observed on imaging were found to have significantly increased odds of developing a postoperative complication compared to individuals with No-AAC. Although controlling for various demographics, lifestyle factors, comorbidities, and medical history, may have attenuated the association between any presence of AAC and complications, this relation remains of clinical significance. One plausible explanation for worse postoperative medical outcomes in patients with AAC could be due to altered hemodynamic blood flow to vital organs due to stiffening of the abdominal vasculature from the calcification [40].

Patients with moderate AAC severity were found to be at 3.48 times higher odds of developing a complication following MIS-ATP lumbosacral fusion. The extensive AAC severity group lost its significance when controlling for confounders. Individuals with low AAC severity had the lowest odds of a complication among the 3 groups despite not reaching significance in both simple and multivariable regression. Interestingly, the moderate AAC severity group had higher odds of complication compared to the extensive AAC severity group. This may be due to a lower sample number of patients in the extensive severity group compared to the moderate group, possibly leading to an inability to accurately detect a significant difference on statistical analysis.

### In the setting of AAC, does the extent of surgery matter?

Our stratified analyses further highlight the interaction between AAC and the number of fused levels and ultimately found that patients with AAC who underwent MIS-ATP fusions of 2 or less levels had increased odds of postoperative complications compared to those patients without calcification (adjusted OR 5.39, 95% CI 1.33–21.90, p = .019). This association was not as direct in patients undergoing 3 or more level fusions, where the adjusted association trended towards, although not reaching, statistical significance (adjusted OR 1.78, 95% CI 0.99–3.18, p = .053). A possible reason for this is that in shorter fusion segments, systemic comorbidities and vessel stiffness from AAC may play a proportionally greater role in postoperative outcomes compared to longer fusions. Additionally, the limited sample size in the ≤2 level group prevented stratification by AAC severity in this specific subanalysis. The reason for this is that the statistical models only converged after collapsing comorbidity, substance use, and race into simplified binary variables, which further highlights the challenges of modeling interactions in smaller strata. These results suggest that the surgical extent and number of fused levels remain important in context when interpreting complication risks in patients with AAC. It is also important to recognize that the average number of fused levels in our MIS-ATP cohort was 2.3 per patient, compared to approximately 1.2 levels per patient in most published TLIF series [41]. This discrepancy reinforces the challenge of direct comparison across approaches, as higher fusion extent inherently predisposes to greater medical complication risk.

### Limitations

To our knowledge, this is the largest and only matched cohort investigation focusing on complication risks in patients with AAC undergoing MIS-ATP fusion surgeries. Additional strengths include the implementation of a validated methodology for the evaluation of AAC extent and the involvement of 3 well-trained graders who reached strong inter-rater reliability, thereby decreasing the chance of human error and grading biases [10]. Additionally, none of the authors’ disclosures were relevant to the study design, implementation, or interpretation of findings. In the matching process, only ASA class and sex were used for matching to avoid overmatching, eliminating the relevance of the AAC phenotype of interest, and collapsing the study sample size. Although the baseline risk profile differences related to AAC are important to control for, they were adjusted through multivariable regression rather than additional matching.

On the other hand, this study is not without limitations. Being a retrospective chart review, this investigation is subject to incomplete and inconsistent documentations. Additionally, operative variables such as intraoperative estimated blood loss and operative duration were not collected. Similarly, clinical variables such as a history of anemia, use of blood thinners at time of surgery, history of bowel disease (IBS, IBD), and postoperative nasogastric tube placement were not collected. These variables may have influenced the rate of complications between the studied groups, but as they were not collected, we cannot state their relevance. Another limitation was the uneven distribution of calcification severity, possibly due to smaller number of patients in low and extensive calcification groups. The statistical significance was lost with multivariate regression, and due to the lack of technical vascular injuries, the study was unable to detect a statistically significant difference between these groups. Also, looking at the incidence of exposurerelated vascular injury, the senior authors’ expertise in MIS-ATP technique introduces a possible selection bias with threat to the external validity as our findings may not be generalizable or reproducible across the wide spectrum of “surgical expertise.” Therefore, additional future multicenter investigations with larger sample size and prospective design are needed to better establish the proportional relationship between the severity of AAC on 1 hand, and the risks of complication on the other.

## Conclusion

The presence of AAC in patients undergoing MIS-ATP fusion was not associated with increased risk of surgical exposure related vascular complications, despite the direct surgical manipulation of the prevertebral vessels and anterior spinal column elongation. Nonetheless, patients with AAC were at a significantly greater risk of developing medical complications following MIS-ATP fusion. Our findings can serve as a patient counseling tool for surgeons performing antepsoas (ATP) and oblique (OLIF) fusions. Accordingly, surgeons can implement preventative strategies to mitigate some of these observed complications and subsequently improve patients’ outcomes and expedite their recovery.

## Authors contribution

**CT:** conception and design, acquisition and data, drafting of the manuscript, critical revision of the manuscript for important intellectual content, administrative/technical/material support, supervision. **HD:** conception and design, acquisition and data, analysis and interpretation of data, drafting of the manuscript, critical revision of the manuscript for important intellectual content.

**RRK:** conception and design, acquisition and data, analysis and interpretation of data, drafting of the manuscript, critical revision of the manuscript for important intellectual content. **RJK:** analysis and interpretation of data, statistical analysis.

**OTZ:** acquisition and data, critical revision of the manuscript for important intellectual content. **SA:** acquisition and data, critical revision of the manuscript for important intellectual content. **AS:** conception and design, critical revision of the manuscript for important intellectual content, administrative/technical/material support. **MNC:** conception and design, drafting of the manuscript, critical revision of the manuscript for important intellectual content, administrative/technical/material support. **TT:** conception and design, acquisition and data, drafting of the manuscript, critical revision of the manuscript for important intellectual content, administrative/technical/material support, supervision.

## Funding

This study did not receive any funding.

## Declaration of competing interest

The authors declare that they have no known competing financial interests or personal relationships that could have appeared to influence the work reported in this paper.
